# Second Update for Anaesthetists on Clinical Features of COVID-19 Patients and Relevant Management

**DOI:** 10.3390/jcm9082542

**Published:** 2020-08-06

**Authors:** Robert P. Weenink, Benedikt Preckel, Abraham H. Hulst, Jeroen Hermanides, Menno D. de Jong, Wolfgang S. Schlack, Markus F. Stevens, Nicolaas H. Sperna Weiland, Markus W. Hollmann

**Affiliations:** 1 Department of Anesthesiology, Amsterdam University Medical Centers, Location AMC, Meibergdreef 9, 1105 AZ Amsterdam, The Netherlands; b.preckel@amsterdamumc.nl (B.P.); a.h.hulst@amsterdamumc.nl (A.H.H.); w.s.schlack@amsterdamumc.nl (W.S.S.); m.f.stevens@amsterdamumc.nl (M.F.S.); n.h.spernaweiland@amsterdamumc.nl (N.H.S.W.); m.w.hollmann@amsterdamumc.nl (M.W.H.); 2 Laboratory of Experimental Intensive Care and Anesthesiology (LEICA), Amsterdam University Medical Centers, Location AMC, 1105 AZ Amsterdam, The Netherlands; 3 Department of Medical Microbiology & Infection Prevention, Amsterdam University Medical Centers, Location AMC, 1105 AZ Amsterdam, The Netherlands; m.d.dejong@amsterdamumc.nl

**Keywords:** COVID-19, SARS-CoV-2, perioperative care, antiviral agents, angiotensin converting enzyme 2, patient-to-professional infectious disease transmission

## Abstract

The COVID-19 pandemic poses great challenges for healthcare workers around the world, including perioperative specialists. Previously, we provided a first overview of available literature on SARS-CoV-2 and COVID-19, relevant for anaesthetists and intensivists. In the current review, we provide an update of this topic, after a literature search current through May 2020. We discuss the evidence on perioperative risk for COVID-19 patients presenting for surgery, the risk of transmission of SARS-CoV-2 in the operating room, and the current literature on laboratory diagnostics. Furthermore, cardiovascular and nervous system involvement in COVID-19 are discussed, as well as considerations in diabetic patients. Lastly, the latest evidence on pharmacological treatment is summarised.

## 1. Introduction

The ongoing COVID-19 pandemic remains a matter of grave international concern. Anaesthetists and other perioperative specialists continue to be confronted with patients who are proven or suspected to suffer from SARS-CoV-2 infection. Recently, we summarised the literature published up until March 2020 to provide an overview of available facts on this disease that are of interest to perioperative specialists [1]. Because of the novelty of this condition, knowledge is rapidly increasing and evolving. Therefore, we performed a new literature search, current through May 2020, to provide the anaesthesia community with an update on relevant perioperative subjects concerning COVID-19. On the basis of discussions within our departments, we identified the most relevant themes for inclusion in this review. PubMed and preprint servers were searched for each topic with appropriate search terms, and articles were manually selected for relevance. As with our previous review, we are aware that our paper has a subjective nature, and that what may today seem true or relevant, may tomorrow prove to be outdated. The current paper should be read as a follow up paper to our previous review. Where new data on subjects discussed in our previous paper were available, this is mentioned in the text. The reader is also referred to another review that is highly relevant for anaesthesia providers during the COVID pandemic [2].

## 2. Perioperative Risk in COVID-19 Patients

The clinical features of COVID-19, which include respiratory deterioration and major inflammatory stress, have been suggested to put surgical patients with COVID-19 at increased risk of perioperative complications [3]. This has recently been confirmed in a cohort study of 1128 patients, mostly from the United Kingdom, United States, Italy, and Spain, undergoing a wide spectrum of elective (25% of patients) and emergency (74% of patients) surgical procedures [4]. All patients in the study were diagnosed with COVID-19, either preoperatively or in the first 30 days after surgery. Of note, 13% of diagnoses were based on radiological or clinical criteria, because laboratory testing for SARS-CoV-2 was not available in these hospitals.

Overall 30-day mortality was 24% and postoperative pulmonary complications (PPC) occurred in 51%, which is much higher than baseline ratios of death and PPC in surgical patients [5]. Even emergency midline laparotomies have lower mortality rates—an international study reports 15% 30-day mortality [6]. In the COVID-19 population, emergency procedures had a mortality rate of 26%, and elective procedures had a rate of 19%. Mortality was higher in men (28%) than in women (18%), and in patients aged ≥70 years (34%) versus patients aged <70 years (14%). Of those 51% of patients who developed PPC, 38% died, compared to 8.7% of those who did not have PPC. Mortality and PPC risk were similar in sensitivity analyses that included only those patients with laboratory confirmed SARS-CoV-2 infection, and only those patients with preoperatively confirmed SARS-CoV-2 infection.

It must be noted that the study carries a risk of selection bias, for instance if COVID-19 diagnosis was missed in less severely affected patients. Nevertheless, even if a significant degree of reporting bias occurred, the results indicate that perioperative infection with SARS-CoV-2 greatly increases perioperative morbidity and mortality. Judging from these data, it may be wise to postpone surgery for all but the most urgent of SARS-CoV-2 positive cases—bearing in mind that it is currently not known how long it takes before perioperative risk has returned to baseline. The fact that a postoperative diagnosis of COVID-19 results in similar death rate and risk of PPC as a preoperative diagnosis indicates that all precautions should be taken to protect postsurgical patients against SARS-CoV-2 infection [2].

## 3. Transmission in the Operating Room

Although the exact transmission routes that SARS-CoV-2 uses are still under debate, several generally accepted assumptions can be made from its epidemiologic characteristics [7] and comparison with related respiratory (corona)viruses [8]. First, as household spread accounts for a large portion of all new infections, it is believed that prolonged close contact is an important factor in SARS-CoV-2 transmission [9]. Second, viral shedding from infected persons occurs predominantly through the production of virus laden respiratory droplets (sized >5 µm) and aerosols (sized < 5 µm) [10,11,12,13]. Additionally, positive reverse transcriptase polymerase chain reaction (rt-PCR) tests were found in faeces and serum, but as rt-PCR detects viable as well as nonviable virus, transmission through these media remains controversial [11,14,15]. Population-based measures to control spread of SARS-CoV-2, such as physical distancing, are not always possible for healthcare workers, especially those working in the operating room (OR). Furthermore, aerosol generating medical procedures (AGMPs), such as endotracheal intubation, airway suctioning, tracheostomy, and bag-mask ventilation are performed regularly in the OR and intensive care unit (ICU) [16,17]. Moreover, theoretical concerns have been raised about the aerosol generating potential of pneumoperitoneum, laser ablation and electrosurgery [18].

During the SARS-CoV epidemic, a high incidence of nosocomial infections among healthcare workers involved in AGMPs was observed. The first reports are now emerging on SARS-CoV-2 transmission to healthcare workers. In Birmingham (United Kingdom) an (unpublished) cross-sectional study found around ~20% seroconversion in working hospital personnel, with an asymptomatic point-prevalence of ~2% [19]. This is in accordance with earlier reports from Italy that suggest a ~20% infection rate among healthcare professionals [20]. Currently, it has been shown that SARS-CoV-2 is present in aerosolised form in the ICU and general wards [12,13]. Furthermore, SARS-CoV-2 remains viable for more than three hours in aerosolised form [21]. On the other hand, several uncertainties surround the discussion on aerosol transmission of SARS-CoV-2. For example, it is unknown which medical procedures have the greatest aerosol generating potential or which aerosol size is most contagious. Additionally, no study confirms viable virus shedding in aerosolised form from awake patients or from pneumoperitoneum, laser ablation or electrosurgery [22,23].

To minimise the risk of infection, the World Health Organization recommends airborne precautions during AGMPs in patients with COVID-19 [24]. Apart from adequate personal protective equipment, such as goggles, gloves, and filtering facepiece respirators 2 (FFP 2, Europe) or N95 masks (United States), this also involves choosing the proper in-hospital environment to perform AGMPs. Modern ORs are usually equipped with positive pressure ventilating systems that inherently convey a risk of aerosol transportation to adjacent rooms and hallways, and most guidelines have suggested avoiding these rooms for AGMPs [25,26,27]. In the ICU and on wards, it seems rational to admit patients to airborne isolation rooms (single patient rooms with negative pressure hierarchy). ORs are usually ventilated with a high amount of air changes per hour, which facilitates aerosol washout [28]. The safest environment to perform AGMPs probably is a room with high fresh air supply (to obtain quick aerosol washout) combined with negative pressure hierarchy (to prevent aerosol distribution).

## 4. Laboratory Diagnostics

The main method for laboratory diagnosis of COVID-19 during the acute stage of the illness remains detection of viral RNA in respiratory specimens by rt-PCR. Antigen detection assays provide an alternative method for virus detection but these are not recommended at present (despite advantages of speed and convenience) because of low sensitivity [29]. While nasopharyngeal or combined nasal and oropharyngeal swab specimens remain the preferred diagnostic specimens, increasing evidence suggests the use of saliva as a promising and convenient alternative specimen, with detection rates ranging from 85 to 100% compared with nasopharyngeal swabs [30,31,32,33,34]. Some studies even suggest increased sensitivity of saliva compared with nasopharyngeal swab specimens.

The duration of viral RNA detection can last over 20 days after illness onset in hospitalized patients [35]. The viral load peaks around the onset of disease and gradually declines over time, hence sensitivity of rt-PCR may be reduced later in the course of the infection. Therefore, in patients with severe disease such as ICU patients, testing of lower respiratory tract specimens should be strongly considered, as viral RNA may be detected at higher levels and for prolonged periods in such specimens [35,36].

Viral RNA detected by rt-PCR does not necessarily represent replicating infectious virus, particularly during later stages of the infection. In a recent study, virus could not be isolated in tissue culture, as an indication for the presence of infectious virus, from specimens with relatively low viral RNA loads, or from specimens collected later than eight days after symptom onset [37]. Importantly, if confirmed, these results will be helpful in guiding public health policy and informing infection control and occupational health decisions.

Particularly during the later stages of the infection, serological tests can be considered to support a diagnosis of COVID-19 in suspected patients, as antibody responses may have developed at that time. During severe infections, SARS-CoV-2-specific serum antibodies (IgG, IgA) begin to emerge within the first week after symptom onset and are detectable in nearly all patients after 10–14 days [35,38]. In mild infections, detectable antibodies appear later than in severe cases, and seem to remain undetected in a proportion of patients [38]. As titers of antibodies during mild infections are lower, it remains to be determined whether this is owing to limited sensitivities of current assays, whether antibodies may appear later in time, and which titers afford protective immunity. In addition, the longevity of antibody responses and the potential for re-infection are important topics of current and future research.

## 5. Cardiovascular Aspects

**Figure 1:**
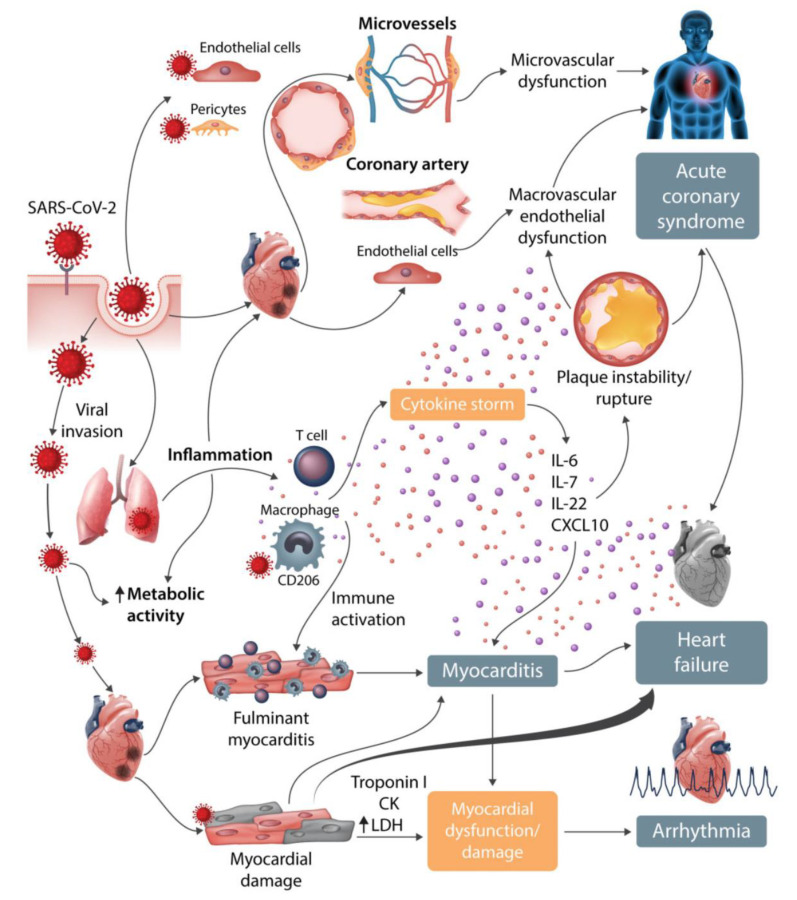
Cardiovascular involvement in COVID-19. Reproduced (with permission) from Guzik et al. [39].

Patients suffering from COVID-19 frequently present with cardiovascular risk factors or cardiovascular disease, contributing to increased morbidity and mortality. The disease itself, or its treatment, however, can also lead to cardiovascular complications (Figure 1). We will first summarise the role of angiotensin converting enzyme (ACE) 2, because of its pivotal role in this disease. Then, we will report the cardiovascular disturbances in COVID-19 and conclude with an overview of coagulation abnormalities in this disease.

### 5.1. The Role of ACE2

**Figure 2:**
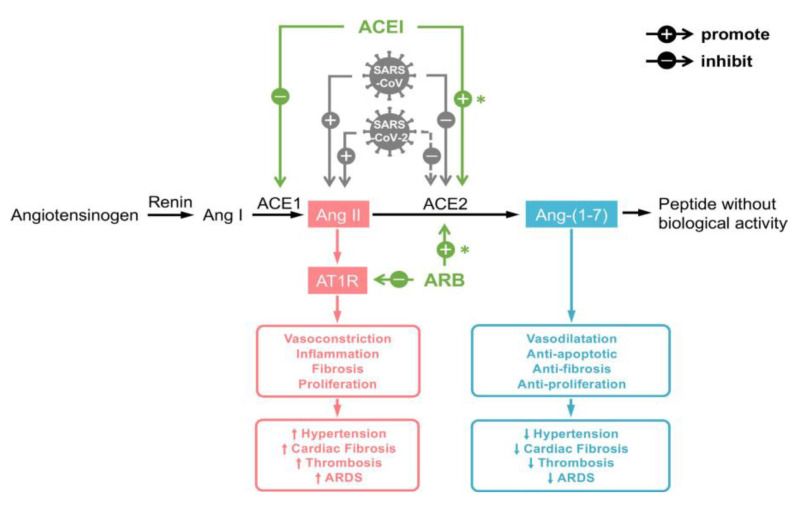
The role of the renin-angiotensin axis in COVID-19. Abbreviations: Ang = angiotensin; ACE = angiotensin converting enzyme; ACEI = ACE inhibitor; ARB = angiotensin receptor blocker; AT1R = angiotensin II receptor type 1, ARDS = acute respiratory distress syndrome. Reproduced (with permission) from Guo et al. [59].

ACE2 is a mostly membrane anchored enzyme, that converts Angiotensin II (AngII) to Ang-(1–7) (Figure 2). A small fraction is shed into the circulation, which is usually not detectable in healthy humans [40]. ACE2 counterregulates the potential harmful effects of activation of the renin-angiotensin axis by decreasing AngII. In addition Ang-(1–7) has been shown to have potential cardioprotective and renoprotective properties [41].

SARS-CoV-2 enters the cell via membrane bound ACE2 and Transmembrane Protease Serine 2 [42]. ACE2 is expressed in many tissues, including the lung, intestines, heart, kidney, and pancreas, explaining the multi-organ involvement in many patients [43,44]. In addition, it is probably overexpressed in the population that seems to be at risk for SARS-CoV-2 infection, including patients with heart failure, hypertension, and insulin resistance [45,46]. SARS-CoV-2 entry into the cell may lead to depletion of membrane bound ACE2 [42,47,48,49]. As ACE2 is the entry receptor for SARS-CoV-2, it has been suggested that patients with an upregulated ACE2 expression are at increased risk of being infected and may have a more serious disease course [50]. In animal studies, use of an ACE inhibitor (ACEi) or angiotensin receptor blocker (ARB) increases ACE2 expression; thus there have been suggestions in scientific and popular literature that the use of these drugs could be harmful [51,52]. These suggestions were, however, not supported by clinical data. Moreover, two recent observational cohort studies published in the New England Journal of Medicine examined the above ideas. Mancia et al. studied the risk of acquiring COVID-19 in a case-control design in 6272 patients with SARS-CoV-2 infection versus 30,759 controls in Italy and concluded that the use of ARBs or ACEis did not increase the risk of acquiring COVID-19 [53]. The findings of this study were confirmed in a second large cohort study and in a case-control study in the Lancet [54,55]. Of course we have to keep in mind that these retrospective studies carry a risk of bias, but the evidence so far does not indicate that withholding these drugs reduces the risk of SARS-CoV-2 infection, or that COVID-19 patients should cease the use of these medications.

Contrary to the suggestion that ARBs and ACEis could be harmful, it has also been suggested that these drugs may in fact be beneficial in COVID-19. Liu et al. showed a correlation between severity of COVID-19 disease course and AngII levels [56]. Previous studies on acute respiratory distress syndrome (ARDS) in ACE2 knock-out mice showed a protective role of ACE2 in preventing lung tissue damage [48]. Lung failure caused by infection of mice with SARS-CoV was reduced by ACE2 [48]. In a meta-analysis of pneumonia studies, patients using ARB/ACEi were less likely to die as compared with patients not taking these drugs [57]. The administration of recombinant ACE2 in piglets with LPS induced lung injury resulted in an immediate decrease of Ang II and TNF-alpha and increase in arterial oxygen tension [58]. It is conceivable that ACEi/ARBs or recombinant ACE2 may serve as a treatment for patients with COVID-19. Indeed, several trials are underway to study this hypothesis.

### 5.2. Cardiovascular Risk Factors and Disease

Nearly three out of four hospitalised COVID-19 patients have at least one co-morbidity, contributing to increased vulnerability. However, it is still unclear whether cardiovascular disease and respective risk factors are causally linked to the disease, or just mirror the increased age of the population [60]. Patients with concomitant cardiac disease have an extremely poor prognosis compared with subjects without a history of cardiac disease, with mortality as high as 36% [61].

Several potential mechanisms have been described [39] that support the hypothesis that both cardiovascular risk factors and disease increase the severity of COVID-19 [62] However, cardiovascular disease can also be a primary phenomenon in COVID-19 patients, owing to the fact that ACE2 is expressed in human heart, vascular cells, and pericytes. Immune system activation along with alterations of immunometabolism may result in plaque instability followed by plaque rupture, contributing to the development of acute coronary events (type 1 myocardial infarction). Thus, ST elevation myocardial infarction may represent the first clinical manifestation of COVID-19. However, in about 40% of COVID-19 patients with ST elevation myocardial infarction, a culprit lesion was not identifiable [63]. Type 2 myocardial infarction (indicative of oxygen supply/demand mismatch) may occur during severe hypoxemia owing to respiratory insufficiency or as a consequence of systemic hypotension: 7–17% of hospitalised COVID-19 patients presented with acute myocardial injury as defined by troponin elevation. In patients with severe infection, evidence of acute myocardial injury was present in up to 32% of patients, and troponin was significantly higher at admission compared with patients with non-severe courses [64].

### 5.3. Myocarditis

As ACE2 is expressed in the cardiovascular system, direct cardiomyocyte infection by SARS-CoV-2 is possible. Myocarditis may appear in COVID-19 several days after initiation of fever. Activated T cells and macrophages infiltrate infected myocardium, resulting in the development of fulminant myocarditis and cardiac damage [39]. Myocarditis should be suspected in patients with COVID-19 and acute-onset chest pain, ST segment changes, cardiac arrhythmia, and haemodynamic instability, especially in those patients without pre-existing cardiovascular disorders.

### 5.4. Arrhythmia

Viral invasion of cardiomyocytes might contribute to arrhythmias. In an early report, 16.7% of COVID-19 patients developed arrhythmias, making this the most prevalent complication after ARDS [11]. Arrhythmia was observed in 7% of patients who did not require ICU treatment and in 44% of subjects who were admitted to an ICU. Individual patients may be at risk of cardiac arrhythmias owing to underlying CVD. Hypertensive patients may have developed left ventricular hypertrophy, leading to increased risk of developing arrhythmia owing to hypoxia [65]. A high frequency of hypokalaemia in patients with severe COVID-19 infection has been reported, probably due to increased urinary loss of potassium (e.g., because of diuretic therapy). Therefore, plasma potassium levels should be monitored.

Amiodarone is the primary antiarrhythmic medication, but combination with hydroxychloroquine and azithromycin should be avoided because of the risk of QT interval prolongation. Special consideration should be given to congenital long QT syndromes in combination with COVID-19 infection, especially if antiviral drugs are applied [65].

### 5.5. Heart Failure and Echocardiographic Changes

Patients with COVID-19 may present with chronic heart failure, and additionally, many factors present in this disease may exacerbate chronic heart failure or lead to acute heart failure. Apart from the cardiac involvement described above (myocardial ischemia, myocarditis, arrhythmias), the acute lung injury itself leads to increased cardiac workload. In fulminant disease, the consequences of ARDS, acute kidney injury, hypervolemia, stress-induced cardiomyopathy, pulmonary embolism, and systemic inflammatory activation (‘cytokine storm’) can contribute to acute heart failure or exacerbation of chronic heart failure [66].

One-third of patients with COVID-19 had normal echocardiography [67]. Left ventricular systolic function was preserved in the majority of patients; the most frequent abnormality was right ventricular dilation or dysfunction. In more severely affected patients, during the course of the disease, further deterioration of the right ventricular parameters was observed, probably related to increased pulmonary resistance [67]. Right ventricular dilation was strongly associated with in-hospital mortality [68], and right ventricular longitudinal strain had a prognostic value in patients with COVID-19 [69].

### 5.6. Extracorporeal Membrane Oxygenation (ECMO)

Veno-venous ECMO can be utilised as an advanced therapy in select patients with COVID-19 related respiratory failure, refractory to traditional critical care management [70]. However, this resource-intensive therapy might be logistically unfeasible in the midst of a pandemic. Early reports showed a very high mortality rate (>90%) of ECMO in COVID-19 [71], but currently, the Extracorporeal Life Support Organisation reports a >55% survival rate in 1338 COVID-19 patients treated with mostly veno-venous ECMO (www.elso.org, accessed 23 July 2020). Thus, in this later phase of the pandemic, ECMO might be a reasonable option in carefully selected patients.

### 5.7. Coagulation Dysfunction

Multiple dysfunctions of the coagulation system occur in patients with COVID-19. The high affinity of the virus for the ACE2 receptor plays a central role in the pathophysiology of these disorders [72,73]. Severe COVID-19 leads to cytokine storm and accelerated cell death, especially in organs infected by SARS-CoV-2. This widespread inflammatory reaction leads to damage of the microvascular system and subsequent activation of the coagulation system with extensive thrombosis in microvessels, especially in the lungs [74]. Recent studies have detected venous thromboembolic events in up 49% of COVID-19 patient [75,76,77]. Not all events were symptomatic, but frequently, a sudden decline in respiratory function indicated a thromboembolic event. In COVID-19 patients suffering from ARDS, pulmonary microthrombi were nine times more prevalent than in patients suffering from influenza-induced ARDS [78]. In sepsis, these processes of immunothrombosis can become overwhelming, leading to sepsis-induced coagulopathy or disseminated intravascular coagulopathy [79]. According to criteria of the International Society of Thrombosis and Hemostasis (ISTH), disseminated intravascular coagulopathy was diagnosed in as much as 71% of COVID-19 non-survivors [80].

The earliest signs of coagulopathy in COVID-19 are increased fibrinogen, D-dimer, and fibrin degradation products. Thromboelastographic testing demonstrates increased clot strength, possibly owing to increased fibrinogen levels [81,82]. Later on, markedly raised D-dimer levels, prolongations in prothrombin time and activated partial thromboplastin time, a mildly reduced platelet count, and hypofibrinogenemia can be observed [80,83,84]. D-dimers above 1 µg/mL at the time of hospital admission have been recognised as an independent risk factor of mortality, with an odds ratio of 18 (95% confidence interval (CI): 2.6–129) [85]. On the basis of these abnormalities in coagulation parameters, several recommendations for coagulation testing in COVID-19 patients have been published. The most recent ISTH interim guidance on coagulopathy in COVID-19 recommends testing D-dimer, prothrombin time, platelet count, and fibrinogen in all patients with COVID-19: a daily check in all admitted COVID-19 patients and daily to twice daily checks in patients who have marked disturbances of coagulation parameters [86].

The role of anticoagulant therapy in COVID-19 remains to be fully determined. The ISTH interim guideline recommends prophylactic dose of low molecular weight heparin in all hospitalised patients in the absence of contraindications [86]. This recommendation is largely based on the observational study of Tang et al. [80]. In the previously cited studies on the incidence of venous thromboembolism, all patients were on low molecular weight subcutaneous heparin thromboprophylaxis, and the use of therapeutic anticoagulation was not associated with an altered all-cause death rate [75,76,77]. An observational study from Mount Sinai, New York showed that therapeutic anticoagulation significantly altered neither overall mortality nor bleeding risk. However, in a subgroup analysis of those patients requiring mechanical ventilation, therapeutic anticoagulation reduced mortality by more than 50% [85]. For clear evidence on higher-dose thromboprophylaxis, we must await the results of ongoing multicentre randomised controlled trials.

## 6. Diabetes

Although probably partly explained by the age of the cohort affected, the prevalence of diabetes in the COVID-19 population seems higher than expected, with a reported prevalence between 5 to 35% [1,87]. Furthermore, patients with diabetes seem more severely affected [1], judging by higher biomarkers of disease and organ failure, longer duration of hospital stay, and increased mortality [88,89,90]. However, diabetes was no longer an independent predictor of mortality after controlling for other comorbidities [88,91].

There is a plausible mechanism to explain the association between the two diseases [92]. As highlighted above, ACE2 is the entry receptor for SARS-CoV-2. As ACE2 is upregulated during hyperglycaemia, this might enable viral cell entry [44,93,94]. Furthermore, ACE2 is present on pancreatic β cells, and as such, the virus might directly affect β cell function. Insulin deficiency following β cell destruction might explain the reported diabetic ketoacidosis on hospital admission, as well as the disproportionally high insulin requirements during hospital stay [93]. In addition to analysing glucose, patients with diabetes should also be screened for diabetic keto-acidosis [93].

### 6.1. Acute Hyperglycaemia

Decline of β cell function not only poses patients with diabetes at risk of glucose dysregulation, it also increases the incidence of new hyperglycaemia without pre-existing diabetes. As such, special attention to blood glucose is warranted for all COVID-19 patients admitted to the hospital, not just those with pre-existing diabetes. To illustrate, one study found a mortality rate of 42% (40 of 96 patients) in patients with new hyperglycaemia, compared with 15% (13 of 88) in patients with pre-existing diabetes [95]. Whether this hyperglycaemia should be regarded as an epiphenomenon (indicator of disease severity) or as a modifiable factor cannot be inferred from these data, and remains part of the ongoing debate of glucose regulation during critical illness [96]. In order to differentiate between acute (stress) hyperglycaemia and unknown diabetes mellitus in a patient with new hyperglycaemia, HbA1c can be measured.

### 6.2. Treatment

The general recommendation for glucose control in ICU patients with diabetes and new hyperglycaemia alike, remains to start a continuous intravenous insulin infusion, with dose adjusted based on frequent glucose monitoring [97]. Physicians should be aware that intensive glucose control is required in COVID-19 patients, because of increased insulin requirements as well as higher incidences of hypoglycaemia compared with other critically ill patients [93,98]. During ICU admission all oral anti-diabetic drugs should be discontinued [93,99], however, the class of dipeptidyl peptidase-4 (DPP4) inhibitors deserves specific attention. Like ACE2, DPP4 was identified as a functional receptor for the spike protein of a subtype of coronavirus (MERS-CoV) [100]. If this pathway is applicable for SARS-CoV-2, the use of DPP4 inhibitors could reduce DPP4 concentrations and as such hinder SARS-CoV-2 cell entrance. However, the clinical relevance of this mechanism and its potential role in treatment of COVID-19 is still unclear, thus cessation of all anti-diabetic drugs except for insulin is still the current recommendation in ICU patients.

## 7. Nervous System Involvement

A growing body of evidence suggests that COVID-19 may affect the nervous system in several ways [101]. Firstly, loss of taste and smell in a large percentage of otherwise paucisymptomatic patients has been reported [102]. This has been hypothesised to be an effect of direct neuroinflammation of cranial nerves, most importantly the olfactory nerve [103]. Secondly, a syndrome of central nervous system involvement may exist, leading to agitation, confusion, executive dysfunction, and corticospinal tract signs in about two thirds of patients admitted to the ICU [104]. This may be the result of meningoencephalitis, as many of these patients show leptomeningeal enhancement on magnetic resonance imaging of the brain [104,105], and a patient with positive rt-PCR for SARS-CoV-2 in cerebrospinal fluid has been reported [106]. The route of transmission has been hypothesised to be transsynaptic—for instance primary infection of the olfactory nerve progressing to central brain areas [103]. However, hematogenic transmission may also be possible [101,107]. Neuroinflammation due to the cytokine storm that occurs in severe COVID-19 has also been suggested [108]. Some authors have hypothesised that involvement of brainstem nuclei, most importantly the nucleus tractus solitarius, could be partly responsible for the ventilatory disturbances seen in COVID-19 patients [103,107]. Another way in which the nervous system may be involved is through cerebral infarction, caused by the hypercoagulability seen in COVID-19 (see above). Ischemic stroke was reported in 2.8% of 214 hospitalised patients in Wuhan (and in 5.7% of severe cases) [109], and in 2.7% of ICU patients in a study from The Netherlands [75].

The implications of nervous system involvement on the management of COVID-19 are currently unclear, but may for instance have implications for the choice of medications, as drugs used in COVID-19 vary in regard to their ability to penetrate the blood-brain barrier [110].

## 8. Pharmacotherapy

Pharmacologic treatment strategies for SARS-CoV-2 infected patients are still lacking robust trial evidence. Several drugs have been proposed and are actually used in the treatment of COVID-19. The interested reader may be referred to two recently published comprehensive reviews on this topic [111,112].

### 8.1. Antiretroviral Drugs

A recent systematic review assessed the effect of the combination of lopinavir and ritonavir in patient with COVID-19. Overall, evidence from randomised trials suggests no clinical benefit in severe COVID-19, or prevention of infection among patients at high risk of acquiring the disease. Data from observational studies were inconclusive [113]. In a randomised, open-label trial in adults with severe COVID-19 [114], patients treated with lopinavir/ritonavir did not show a faster time to clinical improvement compared with patients treated with standard care. Although 14 of the 99 lopinavir/ritonavir-treated patients died within 28 days compared with 25 of the 100 patients in the control group, this difference was not statistically significant in intention-to-treat analysis. Worth mentioning, for those patients treated within 12 days of symptom onset, a faster clinical recovery and a lower mortality was observed. Twenty-one patients with mild to moderate COVID-19, randomised to lopinavir/ritonavir (200 mg/50 mg twice a day) did not show any benefit in terms of time to viral clearance (rt-PCR negativity) or progression to severe disease, compared with 16 patients treated with the antiviral drug ardibol and seven patients without antiviral therapy [115]. In an open-label, randomised, phase 2 trial in patients with mild to moderate COVID-19, the effectivity and safety of a combination of interferon beta-1b, lopinavir/ritonavir, and ribavirin (86 patients) was superior to lopinavir/ritonavir alone (41 patients) with regard to the duration of viral shedding and length of hospital stay [116].

### 8.2. Remdesivir

Compared with our first overview of COVID-19 [1] the position of remdesivir for treatment of COVID-19 patients has not substantially changed. Two randomised clinical trials yielded contradictory results. In a double-blinded randomised trial in 1063 seriously ill COVID-19 patients, remdesivir significantly fastened clinical recovery. While in the control group the course of disease stretched out over 15 days, patients with remdesivir therapy needed only 11 days to recover. A numerical but non-significant lower 14-day mortality of 7.1% versus 12% for remdesivir treated patients was reported [117]. In contrast, in a second double-blinded randomised trial in 237 severely COVID-19 infected patients, remdesivir did not speed up the time to clinical improvement. Although overall no difference was found regarding adverse events between the remdesivir and the placebo group, 12% of remdesivir-treated patients compared with 5% of patients in the placebo group stopped the study drug prematurely owing to experiencing adverse events [118]. This latter study was criticised, as remdesivir was suggested to be started too late in the disease process. In summary, evidence on the effectivity of remdesivir is sparse and conflicting. When started early in the course of the disease, outcome might be improved. Given its acceptable risk profile remdesivir could be a treatment option for severely ill COVID-19 patients.

### 8.3. Chloroquine (CQ) and Hydroxychloroquine (HCQ)

Both CQ and HCQ are seen as promising treatment options, because of their suggested anti-inflammatory and antiviral properties. However, high quality studies demonstrating their effectiveness in COVID-19 are lacking. A retrospective observational study of HCQ in 1376 hospitalised patients with COVID-19 revealed no significant association between HCQ use and the composite endpoint of intubation or death in a time-to-event analysis (hazard ratio (HR): 1.04, 95% CI: 0.82-1.32) [119]. In a retrospective multicentre cohort study of 1438 hospitalised COVID-19 patients, the association between use of HCQ, with or without azithromycin, and clinical outcomes as well as safety was determined. Compared with patients receiving neither drug, treatment with HCQ, azithromycin, or both, was not significantly associated with differences in in-hospital mortality [120]. CQ and HCQ have potentially serious side effects. For instance, HCQ causes QT interval prolongation, especially when used in combination with azithromycin [121,122]. In the abovementioned cohort study, cardiac arrest was significantly more likely in patients receiving HCQ + azithromycin compared with HCQ or azithromycin alone [120]. In conclusion, although various studies and treatment algorithms recommend the use of CQ and HCQ alone or in combination with azithromycin, others suggest that regular use cannot be recommended, based on lack of solid effectivity data and the potential for harm [123].

### 8.4. Corticosteroids

Corticosteroids are used to decrease the host inflammatory response in the lungs, which may induce acute lung injury and ARDS. In a retrospective cohort study including 201 patients with COVID-19, treatment with methylprednisolone, for those patients who developed ARDS, was associated with less mortality (23/50 [46%] with steroids versus 21/34 [62%] without; HR, 0.38 [95% CI: 0.20–0.72]) [124]. On the basis of a most recently published randomized controlled, open-label trial including 2104 COVID-19 hospitalized patients who received oral or intravenous dexamethasone (6 mg once daily for up to ten days) and 4321 with usual care, dexamethasone resulted in lower 28-day mortality among those receiving invasive mechanical ventilation or oxygen alone at randomization, but not among those receiving no respiratory support [125].

### 8.5. Immunomodulatory Agents

In light of the underlying pathophysiology of lung injury or organ failure in general in COVID-19 patients, immunomodulatory agents might combat the amplified immune response and cytokine storm [126].

#### 8.5.1. Tocilizumab

Interleukin-6 seems to play a crucial role in the dysregulated inflammatory response in COVID-19, therefore tocilizumab, a monoclonal antibody directed against interleukin-6, might improve clinical outcome. Twenty-one patients with COVID-19 were treated with 400 mg tocilizumab (most patients received just one single dose). Tocilizumab was associated with improved respiratory function, rapid defervescence, and successful discharge in 91% of patients [127], but the uncontrolled nature of this observation limits the interpretation of the drug’s specific effect and side effects. Thus, more rigorous data are urgently needed; several trials are currently being performed.

#### 8.5.2. Anakinra

The use of the interleukin-1 receptor blocker anakinra might deserve further consideration for clinical testing and possibly treatment of hyperinflammation in patients with COVID-19 related ARDS. In a retrospective study in 29 patients with moderate to severe ARDS and hyperinflammation who were non-invasively ventilated, 10 mg/kg anakinra added to treatment with HCQ and lopinavir/ritonavir was associated with cumulative survival of 90% at 21 days, compared with 56% in 16 patients receiving standard treatment. In addition, after 21 days of follow-up, 21 (72%) patients in the anakinra group, compared with only eight (50%) patients in the standard treatment group showed improved respiratory function [128].

### 8.6. Convalescent Plasma

More than a century old and always in fashion when infectious disease outbreaks occur in the absence of vaccines, treatment with antibody-rich convalescent plasma could be seen as giving the recipients’ immune system a running start. Unfortunately, most what is known about convalescent plasma is founded on reports in which every patient received plasma transfusion. During the 2003 SARS-CoV outbreak in Hong Kong, a group of 80 patients treated with convalescent plasma had a lower mortality rate (10/80 [12.5%]) than overall SARS-CoV-related mortality for admitted patients (299/1755 [17/0%]) [129]. A preliminary report in five critically ill COVID-19 patients with ARDS, reported improvement in all patients after transfusion of convalescent plasma [130]. Similar findings were reported in ten severely ill COVID-19 patients, who all recovered after transfusion of convalescent plasma at a median of 16.5 days after symptom onset [131]. Randomised studies are needed to further define the role of convalescent plasma in COVID-19.

### 8.7. Analgesics and Anti-Febrile Drugs

Apart from specific pharmacological regimens used or studied to treat COVID-19, general supportive therapy is of vital importance. Acetaminophen is the first line antipyretic drug of choice. Despite a large body of literature discussing the pros and cons of nonsteroidal anti-inflammatory drugs in this disease, current recommendation is to not withhold these medications when they are indicated [132].

### 8.8. Summary

To summarise, no pharmacological magic bullet to prevent or treat SARS-CoV-2 infection currently exists. The most solid evidence for a beneficial effect in the treatment of COVID-19 pertains to remdesivir. Immunomodulators such as dexamethasone may be useful to counteract the inflammatory response, but more data are needed. A combination of interferon beta-1b, lopinavir–ritonavir, and ribavirin is a promising approach and seems to be superior to treatment with antiretroviral drugs alone. CQ and HCQ might possess antiviral activity against SARS-CoV-2, however, their use is limited by their narrow therapeutic window and arrhythmogenic potential. Convalescent plasma is an old approach to tackle infectious diseases, however, its effectivity and safety cannot be determined given the lack of randomised controlled trials.

## 9. Conclusions

In this review, we have aimed to provide a timely overview of the literature on SARS-CoV-2 and COVID-19, as far as it is relevant for perioperative specialists. Hopefully, it will enable clinicians to make informed decisions in their daily practice, keeping in mind that evidence continues to evolve rapidly. We will continue to monitor the published literature and will provide a new overview if warranted.
